# Effective Interventions in the Treatment of Self-Harming Behavior in Children and Adolescents with Autism Spectrum Disorder: A Review

**DOI:** 10.3390/children12091184

**Published:** 2025-09-05

**Authors:** Pamela Labarca, Cristian Oyanadel, Melissa González-Loyola, Wenceslao Peñate

**Affiliations:** 1 Department of Psychology, Faculty of Social Sciences, Universidad de Concepción, Concepción 4030000, Chile; elabarca@udec.cl; 2 Deparment of Sociology, University of Groningen, 9712 CP Groningen, The Netherlands; m.a.gonzalez.loyola@rug.nl; 3 Department of Clinical Psychology, Psychobiology and Methodology, Universidad de La Laguna, 38200 Santa Cruz de Tenerife, Spain; wpenate@ull.es

**Keywords:** autism spectrum disorder (ASD), self-injurious behavior, intervention strategies, applied behavior analysis (ABA), cognitive behavioral therapy (CBT)

## Abstract

**Background/Objectives**: Autism spectrum disorder (ASD) is frequently associated with self-injurious behaviors, posing significant risks to individuals and considerable challenges for families and professionals. While various interventions have been proposed, evidence regarding their relative effectiveness remains fragmented. The general aim of this study was to perform a narrative review to analyze effective non-pharmacological interventions targeting self-injurious behaviors (SIBs) in autistic children and adolescents, addressing the following research question: Which non-pharmacological interventions are effective in reducing self-injurious behaviors in autistic children and adolescents, and under what conditions? The review focused on identifying treatment types, contexts of implementation, and outcome efficacy. **Methods**: This review was conducted based on a search in WoS, SCOPUS and PubMed databases. According to the PICOS criteria, we included studies involving children and adolescents with ASD and interventions for self-injurious behaviors. We compared different types of interventions and evaluated outcomes in terms of reduction in SIBs. Eligible studies were those reporting quantitative or qualitative outcomes on SIB interventions, published within the past 10 years. **Results**: Thirteen studies met the inclusion criteria. The interventions included applied behavior analysis (ABA), cognitive behavioral therapy (CBT), sensory integration therapy, and pharmacology. The reported outcomes generally indicated reductions in the frequency and severity of self-injurious behaviors. However, many studies lacked long-term follow-up data, and few addressed the generalization of treatment effects. Methodological variability limited both the comparability across studies and the generalization of results. **Conclusions**: This review emphasized a multidisciplinary, individualized approach to treating self-injurious behaviors in autistic youth. ABA emerged as the most effective intervention, while CBT proved beneficial for higher-functioning adolescents, and sensory therapies addressed specific challenges. Combined treatments showed promise, and family involvement and long-term research remain essential.

## 1. Introduction

Autism spectrum disorder (ASD) is a neurodevelopmental condition that affects a significant proportion of the child and adolescent population. It is considered a spectrum in recognition of the fact that there is no single pattern that typifies autism; rather, there are multiple forms that involve different cognitive abilities, learning styles, and relationships with the environment. However, these different forms share impairments in multiple domains, including social interaction, communication, and behavior. Individuals with ASD often experience challenges in establishing social relationships, exhibit deficits in both verbal and nonverbal communication, and demonstrate repetitive behaviors and rigidity in routines. The severity of symptoms varies widely, underscoring the need for individualized interventions. It is considered a neurodevelopmental disorder as it involves alterations in the development of the central nervous system that manifest from the earliest stages of life, generally before the age of three. These alterations affect the way the brain processes information, which influences the development of social, communicative, cognitive, and behavioral skills [1].

According to the Diagnostic and Statistical Manual of Mental Disorders, fifth edition (DSM-5) [2], ASD is defined by persistent deficits in social communication and interaction, as well as restricted, repetitive patterns of behavior, interests, or activities. The diagnostic criteria require at least two of the following manifestations: (1) difficulties with verbal and nonverbal communication, the use of objects, or movement; (2) rigid or ritualistic behaviors; and (3) altered sensory sensitivities, including hypersensitivity or hyposensitivity, a new feature included in the DSM-5. The DSM-5 also introduces three severity levels, guiding intervention based on the extent of support needed [3].

Among the behaviors associated with ASD, self-injurious behaviors are especially prevalent and represent a critical challenge in both clinical management and daily caregiving. These behaviors, which include head banging, biting, scratching, or other harmful actions toward one’s own body, not only affect individuals’ physical health and emotional well-being but also negatively impact their social development and the quality of life of their caregivers and families. Furthermore, self-injury represents a serious risk of harm and even death [4].

Although these behaviors may appear purposeless, they are often functional, serving communicative or sensory regulation purposes. Individuals with ASD often resort to self-harm as a way to express their needs, manage stress, or cope with frustration, anxiety, and sensory difficulties. Self-harm is any act in which a person deliberately harms themselves [4], and in the context of autism, non-suicidal self-harm is one that causes physical harm, but without suicidal intent. People on the autism spectrum tend to perform these behaviors rhythmically, repetitively, and compulsively, generally in response to states of stress or sensory overload [2]. Although these behaviors can cause pain, individuals with ASD often experience a sense of calm and control after performing them, which reinforces the repetition of these behaviors in situations of emotional or sensory overload.

This complexity makes their management and control even more difficult. The presence of self-injurious behaviors can generate profound distress, sensory overstimulation, and significant physical injuries, which increases the difficulty in treating children and adolescents with ASD [5].

### 1.1. Self-Injurious Behaviors in Children and Adolescents with ASD

Self-biting: Biting is a common form of self-injury, where children bite themselves, usually on their hands, arms, or other accessible body parts. This behavior can range in severity from minor bites to deep wounds [6].

Head banging: This is one of the most severe types of self-injurious behaviors and may involve banging the head against hard surfaces, such as walls or the floor. These behaviors can endanger the child’s physical safety, causing serious injuries [7].

Skin scratching or picking: Children and adolescents with ASD often scratch or pick at parts of their bodies, especially during times of distress, frustration, or sensory overload. This type of self-injury can result in visible injuries, such as marks or wounds [7].

Finger or nail biting: This type of self-injury is more common in those with less severe forms of ASD, although it can also occur in those with more severe disorders. It involves the repetitive action of biting the nails or fingers, sometimes to the point of pain or bleeding [8].

Tearing clothing or ripping skin: In some cases, children with ASD repeatedly strip or rip their clothing, which can be an indirect self-injurious behavior, as it may involve breaking the skin through excessive handling or compulsive tearing of clothing [9].

Swallowing dangerous objects: Some children with ASD may swallow non-food objects as part of self-injurious behavior. Although this is not always traditionally classified as self-injury, it is a dangerous repetitive behavior that can lead to internal injuries [10].

### 1.2. Prevalence and Relevance of the Problem

Once considered rare, ASD is now recognized as a common condition. According to Spain’s National Institute of Statistics (INE), the prevalence rate is 1.94 per 100 individuals, 3.66 in males and 0.75 in females [11]. Worldwide, ASD is a common condition, with a global trend of approximately 1 in 160 children diagnosed with some degree of autism [12].

Given the growing number of affected individuals, the proper management of behaviors associated with ASD has become a significant challenge. Therefore, scientific research is crucial to address this problem, providing evidence-based interventions and techniques that contribute to improving the quality of life of people with ASD and reducing the impact of these behaviors on their environment.

### 1.3. Treatment

Addressing self-injurious behaviors in individuals with ASD is a complex clinical priority. A range of therapeutic approaches, including behavioral, pharmacological, sensory, and family-based interventions, can be used. However, there remains no clear consensus on the most effective treatment, justifying a comprehensive review of the available evidence [11]. A functional behavioral assessment is a key initial step to identify triggers and functions of self-injury, thereby informing individualized and context-sensitive interventions [1].

From a neurodevelopmental perspective on ASD, treatment of self-injurious behaviors is based on learning processes, cognitive changes, and emotional regulation. This implies that, regardless of the specific emphasis of every intervention program, all of them theoretically assume that a given treatments is able to modify the initial neurological vulnerability.

The interventions supported by research include naturalistic developmental behavior interventions (NDBIs), joint attention training, early intensive behavioral intervention, social skills training, music therapy, structured physical activities, and communication-based therapies, such as the Denver model [11,13]. Although these interventions primarily focus on fostering social, communication, and adaptive skills, they can also play an important role in reducing self-injurious behaviors, given that these are often associated with difficulties in emotional regulation, communication, and social interaction. For example, the Denver model and programs focused on social communication can help children and adolescents with ASD express their needs more effectively, thereby reducing the likelihood that they will resort to self-injurious behaviors as a way to manage frustration or feelings of misunderstanding. Likewise, interventions such as music therapy and physical activities can promote emotional regulation and stress management, which are key factors in reducing these behaviors [14].

Several studies have shown promising results in reducing self-injurious behaviors in individuals with ASD through cognitive techniques such as cognitive restructuring, distraction and relaxation training, and the use of visual aids [11]. Behavioral strategies have also been used, including extinction, differential reinforcement, behavioral interruption, behavior redirection, physical or verbal blocking, and reducing aversive stimuli [11].

Additionally, approaches such as applied behavioral therapy, cognitive behavioral therapy, sensory management, and interventions to reduce stress and promote emotional and communicative support have been shown to be useful in this context [15].

As can be seen, the techniques used are diverse, reflecting the complexity of this disorder. The scientific literature on this topic is extensive, as it is a crucial issue that requires effective intervention to prevent serious consequences, such as the risk of death in children and adolescents with ASD.

### 1.4. Review of the State of the Art

When reviewing research on interventions that have shown positive results in managing self-injurious behaviors in individuals with ASD, it is essential to highlight the importance of a multidisciplinary approach. Given the highly complex nature of ASD cases, interventions must involve professionals from diverse fields, such as neurologists, psychiatrists, psychologists, occupational therapists, educational psychologists, speech therapists, nutritionists, teachers, among others.

Self-injurious behaviors in children with ASD have been the subject of extensive research in recent years, and various therapeutic approaches have been studied, such as cognitive behavioral therapy (CBT), applied behavior analysis (ABA), pharmacological treatments, sensory integration therapy, and family-based interventions. Pharmacological treatment has been indispensable for managing self-injurious behaviors, although the variability in response and both the short- and long-term side effects suggest that it should be used with caution [10]. The main pharmacological treatments include atypical antipsychotics (such as risperidone) and selective serotonin reuptake inhibitors (SSRIs), which have been shown to be effective when self-injurious behaviors are associated with comorbid disorders such as anxiety or aggression. However, the lack of long-term studies limits their recommendation as a first-line treatment.

ABA has also proven to be an effective intervention in reducing self-injurious behaviors, especially when applied early, and when it is based on a systematic approach that includes positive reinforcement and behavior modification, there are positive long-term results [8,16].

CBT has also been identified as effective in treating self-injurious behaviors in adolescents with ASD, helping them develop coping skills and reducing anxiety, a factor commonly linked to the onset of these behaviors [17]. Through cognitive and behavioral interventions, such as the recognition of thoughts and emotions, the use of visual support, role-playing games and social stories, and cognitive restructuring and training in relaxation techniques, the frequency and intensity of self-injurious behaviors in children and adolescents with ASD can be reduced [18].

Sensory integration therapy has shown promising results in helping children with ASD regulate their sensory systems, which can reduce self-injurious reactions resulting from sensory overload. This approach complements both pharmacological and cognitive behavioral treatment, as it considers sensory needs and trains functional self-regulation skills [19]. By implementing controlled sensory strategies, the need for self-stimulatory behaviors can be reduced, while at the same time practitioners can work with children in a playful and motivating way to improve other skills, such as communication and executive functions. Furthermore, this approach has the advantage of having no side effects.

Family-based interventions have also been shown to be effective. Training parents to manage self-injurious behaviors within the context of a functional and preventive approach has shown good results [20]. Involving families in the therapeutic process allows for the identification and management of triggers for self-injurious behaviors, in addition to teaching children adaptive alternatives [20]. Training in social skills and emotional self-management has also been key to long-term success.

In summary, the management of self-injurious behaviors in children and adolescents with autism spectrum disorder (ASD) can be understood within a neurodevelopmental framework that emphasizes the dynamic interaction of biological, cognitive, emotional, and environmental factors. The available interventions are diverse and include behavioral, psychoeducational, sensory, and pharmacological approaches, each with specific benefits and limitations. Ongoing research remains essential to improving outcomes and quality of life for individuals with ASD, underscoring the importance of personalized and developmentally informed applications as a cornerstone of effective management.

### 1.5. Limitations and Gaps

Despite advances in research on interventions for managing self-injurious behaviors in individuals with ASD, there are several limitations that should be considered. One of these is the discrepancies in results, as numerous studies have explored the effectiveness of these interventions, but the results are often contradictory, making it difficult to generalize the findings and implement standardized practices [7]. Also, many of the most effective interventions require significant resources, which can make access limited for some families. Furthermore, the cost of therapies, especially behavioral and pharmacological ones, can be an obstacle for its implementation in diverse settings [21].

Regarding gaps, there is a lack of specialized training or professional development, which affects the quality of interventions. Adequate training is essential for professionals to provide effective and up-to-date care [22]. Gaps in cultural and contextual diversity are also observed, as many studies focus on specific contexts, limiting the applicability of results to different settings. It is noted that although some studies suggest benefits of multimodal interventions, there is little research on the effectiveness of combined approaches that integrate behavioral, pharmacological, and sensory therapies. Further exploration is needed on how these approaches can effectively complement each other [16]. A thorough assessment of the deeper causes of self-injurious behaviors, such as sensory overload or emotional difficulties, is often lacking. This lack of understanding limits the personalization of interventions and their adaptation to each patient’s individual needs [20]. Finally, there is a notable lack of longitudinal studies evaluating the durability of intervention effects. Research measuring long-term outcomes is essential to determine the sustained effectiveness of treatments [19,23].

In conclusion, although progress has been made in interventions for managing self-injurious behaviors in ASD, there are several limitations and gaps in current research that need to be addressed to improve therapeutic approaches and ensure more effective, personalized, and accessible care for all affected individuals.

### 1.6. The Current Study

Taking a neurodevelopmental perspective on ASD, this review highlights the capacity of learning processes, cognitive changes, and emotional regulation to alter and modify the behavior of children and adolescents with ASD in relation to their self-harming behaviors. In this sense, this review addressed the research question on effective non-pharmacological interventions targeting self-injurious behaviors in autistic children and adolescents, with a focus on identifying treatment types, contexts of implementation, and outcome effectiveness, through a narrative review of the existing scientific literature. The specific objectives included (1) identifying the main interventions for the treatment of self-injurious behaviors in children and adolescents with ASD; (2) analyzing the effectiveness of interventions based on the reduction in self-injurious behaviors and the general well-being of patients; and (3) determining the limitations and advantages of each type of intervention.

## 2. Materials and Methods

A narrative review of the literature was conducted, in which intervention studies addressing self-injurious behaviors in children and adolescents with ASD were compiled and analyzed. Because the proposed analysis aims to identify effective interventions, it is appropriate to analyze it using the PICOS (Population, Intervention, Comparison, Outcomes, Study design) methodology [24]. Using this methodology, the research question is presented in Table 1:

Different databases (Web of Science (WoS), Scopus, and PubMed) were chosen as the main sources of scientific literature, using the search algorithm shown in Table 2:

The definition of the inclusion and exclusion criteria for research articles is provided in Table 3:

### 2.1. Selection of Studies

**Figure 1:**
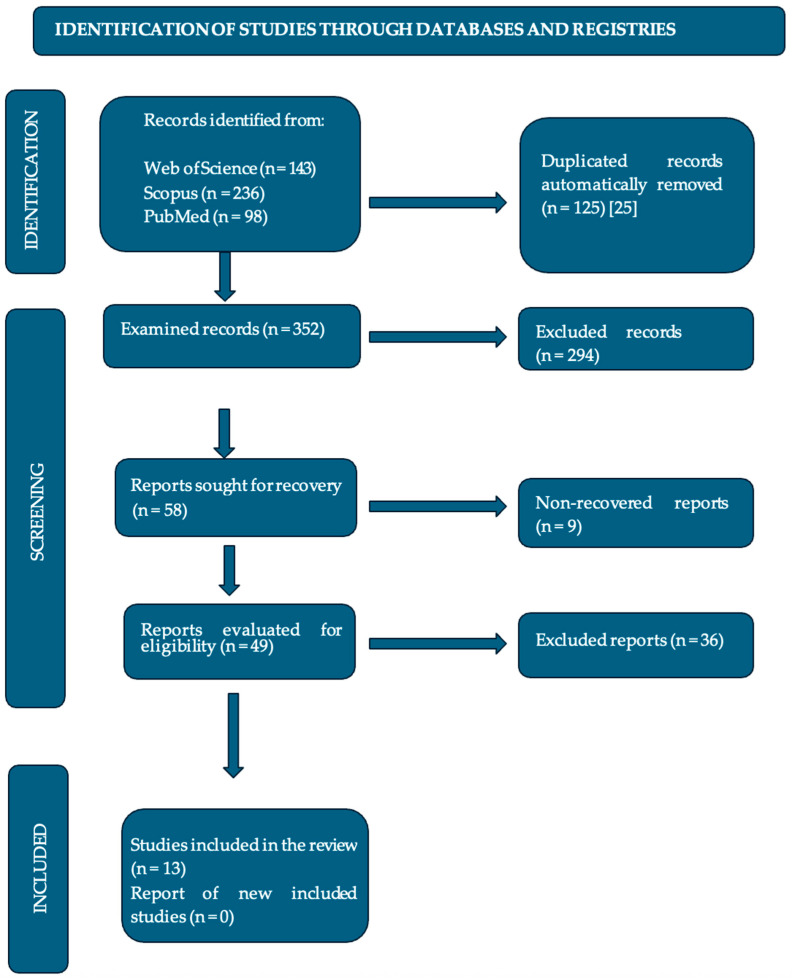
Flowchart of the article selection process (PRISMA, 2020).

In November 2024, the search strategy initially identified a total of 477 studies. After the filtering process, 13 were selected, as shown in Figure 1. These studies included meta-analyses, reviews, randomized controlled studies, experimental studies, case studies, and observational studies.

### 2.2. Extraction of Studies

The articles found in the databases were exported to Zotero [25], a bibliographic reference management software, for storage and organization. Duplicates were identified and removed. Studies were then selected through a filtering process, first by reading the titles and abstracts to identify relevance and then by analyzing the full articles that met the previously defined inclusion and exclusion criteria.

### 2.3. Risk of Bias

Due to the wide variety of methods used in the selected studies (clinical trials, experimental studies, case studies, reviews, etc.), a comparable risk of bias analysis could not be performed across the studies. However, the methodological limitations identified will be discussed.

## 3. Results

### 3.1. Study and Sample Characteristics

Thirteen studies investigating self-injurious behaviors in children and adolescents with ASD, published between 2013 and 2024, were included in the present review, as shown in Table 4.

Most studies were conducted in North America and analyzed populations of children and adolescents diagnosed with ASD, sometimes with comorbidities such as anxiety or behavioral disorders. Samples included individuals with varying levels of ASD severity, including severe cases [16] and psychiatric comorbidities [33]. Most studies focused on school or clinical populations with formal diagnoses, often made using instruments such as the ADOS-2 (Autism Diagnostic Observation Schedule) and other recognized diagnostic tools.

Participants ranged in age from 3 to 18 years. Of the total studies, 12 studies (63.2%) included children aged 3 to 12 years, while 7 (36.8%) included adolescents aged 13 to 18 years. However, the predominant age range was 6 to 12 years, reflecting a greater research concentration on this group.

Sample sizes ranged from fewer than 15 to a maximum of 160 participants. Studies with small samples (fewer than 20 participants) accounted for 31.6%, while medium-sized samples (20–50 participants) represented 26.3%. Large samples (more than 50 participants) comprised 15.8%. This distribution indicates that most studies were based on small and medium-sized samples, which may limit the generalizability of findings.

The predominant methodological design was case studies (31.6%), which focused on personalized interventions and provided a detailed analysis of individual outcomes, often showing reductions of up to 50% in self-harming behaviors. Experimental studies (26.3%) evaluated intervention effectiveness under controlled conditions. Most of these studies reported significant reductions in self-injurious behaviors, typically ranging from 30% to 60%. Randomized controlled trials (26.3%), considered the most rigorous, reported reductions of 30–40% in self-injurious behaviors. Pilot studies (15.8%) tested new approaches in small samples, with reductions in self-injurious behaviors ranging from 30% to 45%.

Various instruments were used to measure the effectiveness of the interventions, tailored to the characteristics of the participants. The most common methods included direct observation, questionnaires, interviews, behavioral logs, and physiological measurements.

### 3.2. Overview of Interventions

The studies primarily addressed therapies aimed at children and adolescents with ASD who exhibit self-injurious behaviors. The most studied approaches were as follows:

Behavioral Therapy (ABA-based interventions): This approach was applied in five studies, in which it was focused on modifying self-injurious behaviors through reinforcement and modeling. ABA showed high levels of effectiveness, with decreases of 40% to 60%, particularly when applied intensively and early [6,16,18,32].

Cognitive Behavioral Therapy (CBT): Three studies used this approach, focusing on treating dysfunctional emotions and thoughts that contribute to self-injurious behaviors. CBT was primarily applied to adolescents, with a focus on emotional regulation and anxiety reduction. Studies reported a significant reduction in self-injurious behaviors of 30% to 50%, especially when emotional factors such as anxiety and negative thoughts were addressed [26,30,34].

Sensory Integration Therapy: Applied in three studies, this therapy aimed to improve sensory processing in children with sensory overload. Sensory integration therapy showed a reduction of up to 40% in self-injurious behaviors, suggesting that improved sensory regulation indirectly contributes to the decrease in these behaviors [9,18,31].

Combined Approaches: Explored in four studies, these integrated ABA with CBT or included medication. They addressed both behavioral and emotional aspects and were associated with substantial reductions in self-injury and improvements in emotional regulation [18,27,29,33].

These therapeutic approaches reflect a diversity of strategies, with most studies focused on CBT and ABA, highlighting their relevance and effectiveness in treating self-injurious behaviors in children and adolescents with ASD.

Overall, the interventions showed significant reductions in self-injurious behaviors. Behavioral therapies produced rapid improvements, while cognitive and occupational therapies yielded notable effects on emotional and sensory regulation. These interventions addressed underlying factors such as sensory overload, emotional difficulties, and coping skills, often requiring a gradual and sustained approach. Although interventions can effectively reduce self-injurious behaviors, the durability of their effects depends on factors such as intervention intensity and long-term follow-up [23]. Evidence regarding their long-term maintenance remains limited, as only a few studies included follow-up measures, and these varied widely in duration.

#### Effectiveness of Interventions

The reviewed studies allowed us to evaluate the effectiveness of different interventions in reducing self-injurious behaviors in children and adolescents with ASD. The findings, grouped by intervention type, are detailed below:

Behavioral Interventions (ABA): These demonstrated the highest effectiveness, with reductions of 50–70% in self-injurious behavior. Individuals experienced a significant reduction in these behaviors, with improvements observed both in the short and long term [6,16,18,32]. This positions ABA as one of the most effective approaches to addressing self-injurious behaviors in individuals with ASD, with enhanced results when combined with emotional approaches such as CBT.

Cognitive Behavioral Therapy: Its effectiveness ranged from a 30% to 90% reduction in self-injurious behaviors. This approach was especially effective in children with high-functioning ASD, as well as in those with high levels of anxiety or stress-related disruptive behaviors [26,30,34]. CBT was one of the most effective interventions, especially in emotional regulation and anxiety reduction.

Sensory Integration Therapy: This showed a reduction in self-injurious behaviors in the range of 30–40%, demonstrating its usefulness in reducing this type of behavior, particularly when these were associated with sensory overload. These interventions stood out in the improvement of sensory self-regulation, which translated into a decrease in disruptive behavioral responses. The effects were more evident in children with hypersensitivity to visual, tactile, or auditory stimuli, allowing them to develop greater tolerance to these stimuli and, consequently, a reduction in self-injurious behaviors. However, its effectiveness depended largely on an adequate assessment of individual sensory needs [9,18,31]. This intervention was moderately effective, making it more appropriate for ASD cases with severe sensory difficulties.

Combined approaches (ABA + CBT, Sensory Integration): Combined interventions, which integrated behavioral and cognitive approaches, proved to be especially useful in addressing both observable behaviors and underlying emotional problems. These interventions offered positive results, achieving significant improvements in reducing self-injurious behaviors and in the management of associated emotional problems, with up to an 85% reduction in self-injurious behaviors [27,29,33]. The use of this intervention is promising for severe cases where self-injurious behaviors have a strong neurochemical and emotional component.

## 4. Discussion

The main objective of this review was to analyze effective interventions for treating self-injurious behaviors in children and adolescents with autism spectrum disorder (ASD) based on the existing scientific literature. Within this framework, three specific objectives were developed: (1) to identify the main interventions, (2) analyze their effectiveness in terms of reducing self-injurious behaviors and the general well-being of patients, and (3) determine the advantages and limitations of each approach.

Regarding the first objective, Table 5 summarizes the main results.

Interpreting the data with caution, due to methodological weaknesses, three main interventions were identified: behavioral interventions based on applied behavior analysis (ABA), cognitive behavioral therapy (CBT), and sensory integration therapy.

Behavioral interventions emerged as the most consistently effective approach for reducing self-injurious behaviors in children with ASD, particularly when tailored to the child’s specific characteristics and needs [6,16,18,27,28,32]. These interventions often involve reinforcing alternative behaviors, modifying environmental triggers, and teaching adaptive coping skills. CBT also demonstrated promising outcomes, especially among children with higher cognitive and language abilities, by enhancing emotional regulation and reducing the frequency of self-injurious behaviors [26,30,34]. These results suggest that CBT may be particularly suitable for a subset of individuals with ASD who can engage with cognitively mediated strategies.

Complementary interventions targeting sensory processing and physiological regulation also showed beneficial effects. Sensory integration therapy led to improvements in self-regulation and reductions in self-injurious behaviors, particularly in children with sensory modulation difficulties [9,32]. Moreover, the integration of pharmacological treatments, such as risperidone and riluzole, were associated with decreases in irritability and emotional dysregulation, which indirectly contributed to the reduction in self-injury [29,33]. Collectively, these findings underscore the value of a multimodal and individualized treatment approach, combining behavioral, cognitive, sensory, and pharmacological strategies as appropriate to each child’s profile, in order to effectively address the complex and multifaceted nature of self-injurious behaviors in autism.

Regarding the second objective, analyzing the effectiveness of interventions for reducing self-injurious behaviors in children and adolescents with ASD, the results highlight considerable variability in outcomes, largely shaped by individual characteristics such as cognitive level, sensory profile, and emotional comorbidities, as well as by the contexts in which interventions are implemented. Behavioral interventions, particularly those grounded in ABA, consistently emerged as the most effective. Several studies reported significant reductions in the frequency and intensity of self-injurious behaviors, often accompanied by improvements in communication and adaptive functioning [6,16,32].

CBT also demonstrated efficacy, especially in adolescents with higher cognitive and verbal abilities. By targeting underlying emotional issues such as anxiety, frustration, and impulsivity, CBT complements behavioral strategies and broadens the therapeutic scope [26,30,34]. These findings support the integration of CBT into comprehensive intervention plans, particularly for adolescents with sufficient cognitive resources.

Sensory and occupational therapies demonstrated moderate effectiveness, primarily by addressing sensory overload and enhancing self-regulation. However, this conclusion should be interpreted with caution, as the two supporting studies are essentially case studies. Additionally, in the case of sensory therapy, no objective data (such as observed changes on a sensitivity scale) were provided to confirm improvements in sensory sensitivity [9,10,11,12,13,14,15,16,17,18,19,20,21,22,23,24,25,32,33]. These approaches appear especially relevant when self-harm is driven by sensory dysregulation. In more severe cases, particularly those with significant psychiatric comorbidity, pharmacological interventions may be warranted [29,33]. Pharmacotherapy is therefore seen as a complementary strategy, provided it is administered under rigorous medical supervision.

Taken together, the evidence suggests that behavioral (especially ABA) and cognitive behavioral interventions hold the strongest support for effectiveness in reducing self-injury among youth with ASD. Sensory therapies offer valuable support for children with sensory-related challenges, and pharmacological treatment plays a supplementary role in complex or treatment-resistant cases. Ultimately, these findings reinforce the importance of multimodal, individualized treatment plans that incorporate behavioral, emotional, sensory, and pharmacological components as needed. Family involvement plays a crucial role in sustaining progress and ensuring long-term success, though the level of parental engagement can vary and should be carefully considered. Future research should explore how different combinations of interventions yield differential outcomes depending on each child’s clinical and functional profile.

Regarding the third objective—identifying the advantages and limitations of the reviewed interventions for reducing self-injurious behaviors in children and adolescents with ASD—the findings emphasize that each therapeutic modality brings unique strengths and challenges that must be considered when designing individualized treatment plans. Although the sample sizes were generally small, ABA stands out for its high efficacy, particularly in structured, intensive, and personalized formats. Studies show that ABA can produce significant reductions in self-injury, even in severe cases [6,16]. One of ABA’s main advantages is its flexibility in integrating with other interventions, such as CBT, thereby enhancing treatment outcomes by addressing both behavioral and emotional dimensions simultaneously [32]. However, the implementation of ABA can be resource-intensive, requiring substantial time, specialized personnel, and funding, which may limit its scalability and accessibility.

CBT presents distinct advantages in targeting emotional and cognitive contributors to self-injurious behaviors, such as anxiety, impulsivity, and maladaptive thought patterns. Research supports its utility in adolescents with sufficient verbal and cognitive abilities, highlighting improvements in emotional regulation and reductions in self-harm [30,34]. Nevertheless, the applicability of CBT is limited in younger children or those with lower cognitive functioning, as the intervention relies on abstract reasoning and comprehension of therapeutic techniques, which may restrict its effectiveness in these populations.

Sensory integration therapies offer particular benefits for individuals whose self-injurious behaviors are linked to sensory processing difficulties. These interventions help reduce sensory overload and improve tolerance to environmental stimuli, contributing to better self-regulation and, in some cases, a decrease in self-harm [9,32]. However, the direct effects on self-injury tend to be more modest compared to ABA or CBT, and outcomes may vary depending on the child’s sensory profile. These findings suggest that sensory therapies are most effective when used as a complementary component within a broader, multimodal intervention framework.

Pharmacological interventions, while potentially beneficial in severe cases or those with complex neuropsychiatric comorbidities, also present important limitations. These interventions are useful in reducing irritability and associated behaviors; however, their long-term effectiveness is limited, and side effects are a concern [29,33]. As such, pharmacotherapy is generally recommended as complementary to psychosocial interventions rather than a primary treatment strategy, and its use requires careful medical oversight.

In summary, while ABA and CBT offer some evidence for effectiveness, their success depends on contextual and individual factors, including cognitive functioning and available resources. Sensory therapies and pharmacological treatments play supportive roles within comprehensive, individualized intervention plans. A thoughtful combination of these approaches, tailored to the specific needs and capacities of each child, appears to be the most promising path forward in addressing self-injurious behaviors in youth with ASD.

Behavioral interventions such as ABA and CBT demonstrate the strongest empirical support for reducing self-injurious behaviors in children and adolescents with ASD. ABA, in particular, shows high efficacy even in severe cases, while CBT is especially effective for adolescents with higher cognitive and verbal abilities, targeting underlying emotional contributors such as anxiety and stress. However, the implementation of both interventions requires trained professionals, time, and financial resources, which can limit their accessibility in some contexts. It is also important to consider alternative explanations for interventions that show smaller or no effects. Some therapies may not align with the cognitive, communicative, or sensory abilities of participants; for instance, cognitively demanding approaches like CBT may be less effective in children with limited verbal skills or abstract reasoning abilities. Additionally, interventions that are brief in duration, delivered by insufficiently trained professionals, or inconsistently implemented may yield reduced or null effects, masking the true potential of the method.

Sensory integration and pharmacological therapies serve important complementary roles. Sensory-based interventions are particularly beneficial for individuals with sensory processing challenges, contributing to reductions in self-injury through improved regulation and sensory tolerance. Pharmacological treatments, although not a primary intervention, can be valuable in managing severe cases or those involving complex neuropsychiatric comorbidities, provided that their use is carefully monitored due to the potential for adverse effects.

Overall, the evidence supports a comprehensive, personalized, and multimodal treatment approach that combines interventions according to the specific needs and profiles of each individual. Combining strategies such as ABA and CBT can enhance therapeutic outcomes by addressing both behavioral and emotional domains while integrating sensory and pharmacological support when clinically indicated. Furthermore, interventions supported by information and communication technologies (such as Augmented Reality) have proven effective in improving cognitive and social skills in people with ASD [35]. The application of these technologies combined with the aforementioned effective treatments can increase this effectiveness.

According to the limitations identified in the reviewed studies, several methodological issues remain, despite the promising results of the interventions. The included studies were methodologically heterogeneous, differing in design, intervention types, outcome measures, and sample characteristics. In addition, the number of studies available for each design was relatively small. The predominance of small sample sizes restricts the generalizability of the findings, while the fact that most studies were conducted in the United States highlights a lack of cultural and socio-economic diversity, limiting applicability to broader populations. Although some research considered comorbidities, these factors were not consistently incorporated into intervention designs. Additionally, the scarcity of longitudinal studies makes it difficult to assess the long-term effectiveness of treatments. The wide variety of study methodologies also prevented a precise comparison of risk of bias and methodological quality across studies. As a result, quantitative syntheses such as meta-analyses, including *p*-values, confidence intervals, and effect sizes, could not be performed, restricting the objective evaluation of outcomes.

Taken together, these limitations suggest caution when generalizing the findings beyond Western contexts and underscore the need for future research employing more rigorous designs, larger and more diverse samples, longer follow-up periods, and careful consideration of comorbid symptoms, such as anxiety and obsessive behaviors, given their complex interactions with self-injurious behaviors in children and adolescents with ASD.

As a general conclusion, addressing self-injurious behaviors in children and adolescents with ASD requires a multidisciplinary, individualized, and evidence-based framework. This narrative review offers clinicians and practitioners an updated synthesis of effective therapeutic options, guiding them in selecting interventions that are not only empirically supported but also tailored to each child’s developmental, cognitive, and emotional characteristics. By promoting informed and responsive clinical practices, this review aims to support the sustained improvement in the quality of life for individuals with ASD and their families.

## Figures and Tables

**Table 1:** PICOS model.

Study topic	Narrative review of effective interventions for treatment of self-injurious behaviors in children and adolescents with ASD
Population	Children and adolescents with ASD
Intervention	Interventions for self-injurious behaviors in ASD (behavioral therapy, occupational therapy-based interventions, ABBA method, etc.)
Comparison	Comparison of different types of interventions considering their effectiveness
Outcome	Results of interventions will be evaluated in terms of reduction or decrease in self-harming behaviors, improvements in quality of life, or adaptive behavior
Study design	Controlled clinical trials, cohort studies, and case–control studies. Longitudinal studies and intervention studies with control groups are the most appropriate for evaluating long-term effectiveness of interventions

**Table 2:** Search strategy.

Pattern	WoS	Scopus	PubMed
(“adolescents”) AND (“children”) AND (“autism spectrum disorder”) AND (“self-injurious behaviors”) AND (“Intervention” OR “Psychological Interventions” OR “cognitive behavioral therapy” OR “TCC” OR “Psychotherapy” OR “Clinical Psychology” OR “Intervention program*” OR “intervention*” OR “treatment program*” OR “therapeutic program*” OR “therapy program*” OR “clinical intervention*” OR “clinical treatment*” OR “psychological treatment*” OR “cognitive behavioral therapy*” OR “ABA therapy” OR “behavioral therap*” OR “ABA” OR “occupational therapy” OR “sensory therapy” OR “sensory integration” OR “effective interventions” OR “ABA behavioral therapy” OR “Family based therapy” OR “asperger” OR “self-harm” OR “self-injurious behavior” OR “interpersonal therapy” OR “Cognitive therapy” OR “behavioral therapy).	143	236	98

**Table 3:** Inclusion and exclusion criteria.

Inclusion Criteria	Exclusion Criteria
Children and adolescents (up to 18 years old) diagnosed with autism spectrum disorder. Publication must be dated no more than 10 years ago to ensure the relevance and timeliness of the approaches. Studies evaluating interventions designed to reduce or manage self-injurious behaviors. Includes different types of interventions, such as behavioral, cognitive, sensory integration, and educational therapies, among others. Articles presenting quantitative and qualitative results on the effectiveness of interventions. Research directly addressing interventions to treat self-injurious behaviors in children and adolescents with ASD. Studies that clearly describe the methodology used in the intervention (behavioral, pharmacological, sensory, etc.). Articles that include data on the effectiveness of the intervention. No language restrictions.	Studies that focus exclusively on adults with ASD or populations not diagnosed with ASD. Studies that primarily investigate medical or psychological conditions other than ASD, unless they include a significant subgroup of participants with ASD. Studies that do not specifically evaluate interventions targeting self-injurious behaviors in children and adolescents with ASD. Articles not directly related to self-injurious behaviors in ASD. Studies of other disorders or interventions not applicable to ASD. Research without empirical evidence or studies that have not been peer-reviewed.

**Table 4:** Characteristics of the included studies.

Author(s), Year, Country	Type of Study	Intervention/Therapy	Key Findings
Weiss et al. [26], (2018) Canada	Randomized controlled	Cognitive behavioral therapy	Improvement in emotional regulation and reduction in self-harming behaviors.
Peterson et al. [27], (2024) USA	Experimental	Applied behavior analysis and other behavioral interventions	Reduction in self-harming behaviors and other problematic behaviors.
Alakhzami & Chitiyo [6], (2022) USA	Experimental	Functional communication training	Reduction in self-harming behaviors.
Robinson et al. [28],(2019) USA	Case	Behavioral interventions	Reduction in self-harming behaviors.
Sabus et al. [29], (2019) USA	Review	Pharmacotherapy	Pharmacological strategies; effectiveness of risperidone; pharmacotherapy should be combined with behavioral therapy.
Wood et al. [30], (2019) USA	Randomized controlled	Cognitive behavioral therapy	Significant decrease in both anxiety and self-injury behaviors.
Morano et al. [18], (2017) USA	Meta-analysis	Behavioral treatments	Reduction in self-harming behaviors.
Boesch et al. [16], (2015) USA	Case	Behavioral approach	50% reduction in self-injurious behaviors and effectiveness in severe cases.
Minshawi et al. [31], (2014) USA	Observational	Association analysis	Relationship between self-harm and clinical features; key functional assessment for intervention.
Schaaf et al. [9], (2013) USA	Randomized controlled	Sensory integration therapy	40% reduction in self-injurious behaviors; improvement in self-regulation.
Izurieta Cossio [32], (2023) Peru	Case	Behavioral intervention	Reduction in self-injurious behaviors; effective individualized intervention.
Ghaleiha et al. [33],(2013) Iran	Randomized controlled	Pharmacotherapy	Improvement in irritability and indirect reduction in self-harm.
Oshima et al. [34], (2023) Japan	Randomized controlled	Cognitive behavioral therapy	Improvements in emotional self-regulation and reduction in self-harm.

**Table 5:** Overall effectiveness of therapies.

Type of Therapy	Overall Effectiveness
Behavioral Therapies	Behavioral therapy is highly effective in reducing self-injurious behaviors, especially with applied behavioral intervention (ABA). The best results are seen in young children, although it is also useful in adolescents with moderate self-injurious behaviors. It is especially effective for higher-functioning individuals who can learn new behavioral skills quickly.
Cognitive Behavioral Therapies	Cognitive behavioral therapies show positive effectiveness in terms of emotional regulation, with significant reductions in self-injurious behaviors, particularly in managing frustration and anxiety. They are especially effective in children and adolescents with greater functional and verbal ability who can benefit from problem-solving and the development of emotional coping skills.
Occupational Therapy/Sensory Integration	These therapies are moderately effective, and they are especially useful for people with sensory overreactions. These therapies help reduce self-injurious behaviors related to sensory overload. They are most useful for children and adolescents with sensory difficulties and can also be applied to people with a wider range of functional abilities.
Combined Interventions with Medication	These interventions are effective for severe cases of ASD, especially those with emotional or neuropsychiatric comorbidities. Pharmacological interventions may be necessary in combination with other therapies to manage severe or neurochemical symptoms and are more common in adolescents with severe self-injurious behaviors.
Combined Interventions (ABA + CBT, SI)	These interventions are highly effective in the combined management of behaviors and emotions, offering integrated improvements that address multiple factors. These interventions are ideal for children and adolescents with ASD, especially those with emotional difficulties and severe self-injurious behaviors, who require a comprehensive approach that addresses both behavioral and emotional aspects.
